# Image quality assessment of pediatric chest and abdomen CT by deep learning reconstruction

**DOI:** 10.1186/s12880-021-00677-2

**Published:** 2021-10-10

**Authors:** Haesung Yoon, Jisoo Kim, Hyun Ji Lim, Mi-Jung Lee

**Affiliations:** Department of Radiology and Research Institute of Radiological Science, Severance Hospital, Yonsei University College of Medicine, 50-1 Yonsei-ro, Seodaemun-gu, Seoul, 03722 South Korea

**Keywords:** Pediatric, CT, Image quality, Deep learning, Iterative reconstruction

## Abstract

**Background:**

Efforts to reduce the radiation dose have continued steadily, with new reconstruction techniques. Recently, image denoising algorithms using artificial neural networks, termed deep learning reconstruction (DLR), have been applied to CT image reconstruction to overcome the drawbacks of iterative reconstruction (IR). The purpose of our study was to compare the objective and subjective image quality of DLR and IR on pediatric abdomen and chest CT images.

**Methods:**

This retrospective study included pediatric body CT images from February 2020 to October 2020, performed on 51 patients (34 boys and 17 girls; age 1–18 years). Non-contrast chest CT (n = 16), contrast-enhanced chest CT (n = 12), and contrast-enhanced abdomen CT (n = 23) images were included. Standard 50% adaptive statistical iterative reconstruction V (ASIR-V) images were compared to images with 100% ASIR-V and DLR at medium and high strengths. Attenuation, noise, contrast to noise ratio (CNR), and signal to noise (SNR) measurements were performed. Overall image quality, artifacts, and noise were subjectively assessed by two radiologists using a four-point scale (superior, average, suboptimal, and unacceptable). A phantom scan was performed including the dose range of the clinical images used in our study, and the noise power spectrum (NPS) was calculated. Quantitative and qualitative parameters were compared using repeated-measures analysis of variance (ANOVA) with Bonferroni correction and Wilcoxon signed-rank tests.

**Results:**

DLR had better CNR and SNR than 50% ASIR-V in both pediatric chest and abdomen CT images. When compared with 50% ASIR-V, high strength DLR was associated with noise reduction in non-contrast chest CT (33.0%), contrast-enhanced chest CT (39.6%), and contrast-enhanced abdomen CT (38.7%) with increases in CNR at 149.1%, 105.8%, and 53.1% respectively. The subjective assessment of overall image quality and the noise was also better on DLR images (*p* < 0.001). However, there was no significant difference in artifacts between reconstruction methods. From NPS analysis, DLR methods showed a pattern of reducing the magnitude of noise while maintaining the texture.

**Conclusion:**

Compared with 50% ASIR-V, DLR improved pediatric body CT images with significant noise reduction. However, artifacts were not improved by DLR, regardless of strength.

**Supplementary Information:**

The online version contains supplementary material available at 10.1186/s12880-021-00677-2.

## Background

The need for pediatric computed tomography (CT) examinations is constantly increasing despite the "as low as reasonably achievable" principle and concerns of radiation hazards for children. Pediatric body CT, including in emergency rooms and in tumor patients, is an important imaging test in children. With the development of technology, efforts to reduce the radiation dose have continued steadily, with the development and use of iterative reconstruction (IR) as a typical example.

Over the past decade, the IR algorithm has been used to produce high-resolution images by decreasing image noise through the use of computational processing, resulting in better image quality with lower radiation dose compared with single reconstructed filtered back projection (FBP) in adults [1, 2] and children [3–6]. The recently developed adaptive statistical iterative reconstruction-V (ASIR-V) technique provides a short reconstruction time with better image quality and lowers radiation dose than other IR algorithms [7, 8]. However, ASIR-V still does not overcome excessive image smoothing and unnatural image appearance. Hybrid IR images that blend IR with FBP can be used to decrease this texture problem, although a trade-off between image noise and image texture occurs [9].

Recently, image denoising algorithms using artificial neural networks, termed deep learning reconstruction (DLR), have been applied to CT image reconstruction to overcome the drawbacks of IR while achieving good image quality [10–15]. However, there have been a limited number of studies evaluating this technique in a small number of children and the technique was only evaluated in abdomen CT images [16–18]. The purpose of our study was to compare the objective and subjective image quality of DLR and IR on pediatric abdomen and chest CT images.

## Methods

This was a retrospective study approved by the institutional review board at our institution, and the need for informed consent was waived.

### Study population

We included all consecutive pediatric patients who underwent chest or abdomen CT at our institution between February 2020 and October 2020 with the same CT system (Revolution CT; GE Healthcare), which has a routine protocol including DLR. We retrospectively reviewed 51 patients. There were 34 boys and 17 girls with a mean age of 11.5 ± 4.6 years (range 1–18 years). Non-enhanced chest CT (n = 16), contrast-enhanced chest CT (n = 12), and contrast-enhanced abdomen CT (n = 23) images were included. Height and weight were recorded at the time of CT examination and BMI was calculated. Body weight group was divided as < 20 kg, 20–60 kg, and > 60 kg.

### Phantom study

In general, signal to noise (SNR) and contrast to noise ratio (CNR) are used to measure the amount of noise (magnitude) in images. However, the standard deviation (SD) used in the SNR and CNR calculations has different values depending on the region of interest (ROI) position in the human body image with a non-homogeneous medium, and SNR and CNR only evaluate the noise magnitude. Noise power spectrum (NPS) is a method that can evaluate the magnitude and texture of image noise in the spatial frequency domain [19] and it can overcome the drawbacks of SD measurement in SNR and CNR calculation. For NPS analysis, we scanned the uniformity module of the Catphan 500 phantom (Catphan 500, The Phantom Laboratory, NY, USA), and performed three scans including the dose level of the patient image used in this study. We directly implemented a 3D-based NPS based on the method presented by the American Association of Physicists in Medicine (AAPM) [20], and used Matlab (Version R2017a, The MathWorks, Inc., MA, USA) for this calculation.

### Scanning technique and radiation dose measurements

All patients were examined using a 256-slice CT (Revolution CT; GE Healthcare). Peak kilovoltage (kVp) was divided in to three groups by weight: 100 kVp for > 40 kg, 80 kVp for 15–40 kg, and 70 kVp for < 15 kg. An automatic dose modulation technique (Smart mA; GE Healthcare) was used with a range of 50–200 mAs. The noise index was 33 for abdomen CT and 22 for chest CT. Other parameters used to generate images were as follows: gantry rotation time, 0.35 s; coverage speed, 226.79 mm/s; pitch, 0.992:1; and slice thickness, 2.5 mm.

Weight-based IV contrast injection was used with settings of 1.5–2.0 ml/kg with a maximum of 100 ml, using 300 mg iodine/ml concentration intravenous contrast iobitridol (Xenetix; Laboratoires Guerbet). The contrast was injected through an upper extremity peripheral intravenous line, followed by a saline chaser of 0.5 ml/kg. Injection speed was adjusted for a total injection time of 15 s or less. For contrast-enhanced abdomen CT, a fixed time interval of 60 s after contrast injection for portal phase without bolus tracking was used. For contrast-enhanced chest CT, a circular ROI was placed at the main pulmonary artery and the CT scan began 4 s after the threshold attenuation of 100 Hounsfield units (HU) was reached.

Four axial reconstructions were generated for each patient with a 2.5 mm slice thickness and 2.5 mm slice interval according to the standard algorithm: 50% ASIR-V, 100% ASIR-V, medium- and high-strength DLR (TrueFidelity; GE Healthcare). We set the blending factors to 50% and 100% according to previous experience [3, 4]. DLR provides three selectable reconstruction strength levels (low, medium, and high) to control the amount of noise reduction with a standard reconstruction kernel. We chose medium and high based on our preliminary experience. TrueFidelity is the first clinically available deep learning-based CT reconstruction technique which is based on deep neural network trained with low-dose raw CT projection data. The ground truth data used to train the algorithm were filtered back projection CT images resulting from ideal data acquisition conditions, both from phantoms and patients in a clinical setting. The output is a reconstructed image that appears as if it had been reconstructed from high-dose raw CT data. However, the details about the network architecture and the training process are not publicly available [21].

The CT dose index volume (CTDIvol, mGy) and dose-length product (DLP, mGy × cm) of all patients were recorded in both CT examinations. CTDIvol was converted to size-specific dose estimates (SSDE) based on the American Association of Physicists in Medicine Report 204 [22]. Patient-specific dimensions were obtained from axial CT images at the carina on chest CT and at the main portal vein on abdomen CT. We used the sum of anteroposterior and lateral dimensions to determine patient effective diameter and conversion factors. The following equation was used to calculate the effective dose (ED, mSv): ED = DLP × W_T_ (tissue-weighting factor; variable according to kVp, organ, and age [23]). Tissue-weighting factors of less than 80 kVp are unknown, so a tissue-weighting factor of 80 kVp was adopted for 70 kVp studies.

### Quantitative image analysis

Quantitative analysis of axial images was performed by a board-certified radiologist with 9 years of experience. The mean attenuation (HU) and SD were measured by manually placing the round ROI (8–10 mm in diameter) using a picture archiving and communication system (PACS) workstation (Centricity Radiology RA1000; GE Healthcare) in the mediastinal/soft-tissue window setting (window level, 50 HU; window width, 350 HU). On chest CT images, ROIs were placed in lung and paraspinal muscles at the level of the carina. On abdomen CT images, ROIs were placed in liver, aorta, and paraspinal muscles at the level of the main portal vein on axial images. To obtain reliable measurements for the areas, each ROI was positioned to encompass the homogeneous portion and did not include surrounding structures or vessels. Image noise was defined as the SD of the pixel values obtained from the paraspinal muscle. Both contrast- and signal-to-noise ratios (CNR and SNR) were defined as CNR = |HU_object_ − HU_muscle_|/SD_noise_ and SNR = HU_object_/SD_noise_ [24]. Also, we calculated the NPS peak (HU^2^ mm^2^) and NPS average spatial frequency (mm^−1^) from each NPS curve measured using phantom. The NPS peak shows the magnitude of the noise, and the NPS average shows the texture of the noise.

### Qualitative image analysis

CT images were independently reviewed by two board-certified pediatric radiologists with 17 and 9 years of experience who were blinded to the clinical findings and the CT reconstruction methods. Images were displayed on the PACS in random order and two radiologists independently recorded their opinions on overall image quality, noise, and motion or beam hardening artifacts. A four-point scale was used: 4 was superior, 3 was average, 2 was suboptimal, and 1 was unacceptable.

### Statistical analysis

All statistical analyses were performed using MedCalc software (version 12.1.0; MedCalc Software). Patient demographic characteristics and dose descriptors (CTDI_vol_, DLP, SSDE, and ED) are summarized and presented as the mean and SD. Repeated measures ANOVA with pairwise comparisons and Bonferroni correction were performed to compare the reconstructions concerning attenuation, noise, CNR, and SNR. Wilcoxon signed rank and Cohen kappa tests were performed to compare qualitative evaluation and to assess interobserver agreement. Agreement between reviewers is expressed as κ values: κ values of 0–0.20, 0.21–0.40, 0.41–0.60, 0.61–0.80, and greater than 0.81 indicated poor, fair, moderate, good, and excellent agreements, respectively. A *p*-value of less than 0.05 was considered statistically significant.

## Results

The mean weight and BMI of the patients were 44.3 ± 18.9 kg and 20.3 ± 5.1 kg/m^2^, respectively. Two patients had metallic hardware within the scanned field of view of the CT images. CTDI_vol_, DLP, SSDE, and ED of chest CT images were 1.3 ± 0.5 mGy (range, 0.6–2.5 mGy), 49.0 ± 26.3 mGy × cm (range 16.5–112.9 mGy × cm), 2.0 ± 0.6 mGy (range 1.2–3.1 mGy), and 2.2 ± 3.2 mSv (range 0.7–16.1 mSv), respectively. CTDI_vol_, DLP, SSDE, and ED of abdomen CT images were 1.5 ± 0.6 mGy (range, 0.4–3.2 mGy), 77.9 ± 35.0 mGy × cm (range 12.6–147.7 mGy × cm), 2.5 ± 0.9 mGy (range, 0.8–4.7 mGy), and 2.0 ± 0.7 mSv (range, 0.7–3.7 mSv), respectively.

### Quantitative image assessment

**Fig. 1:**
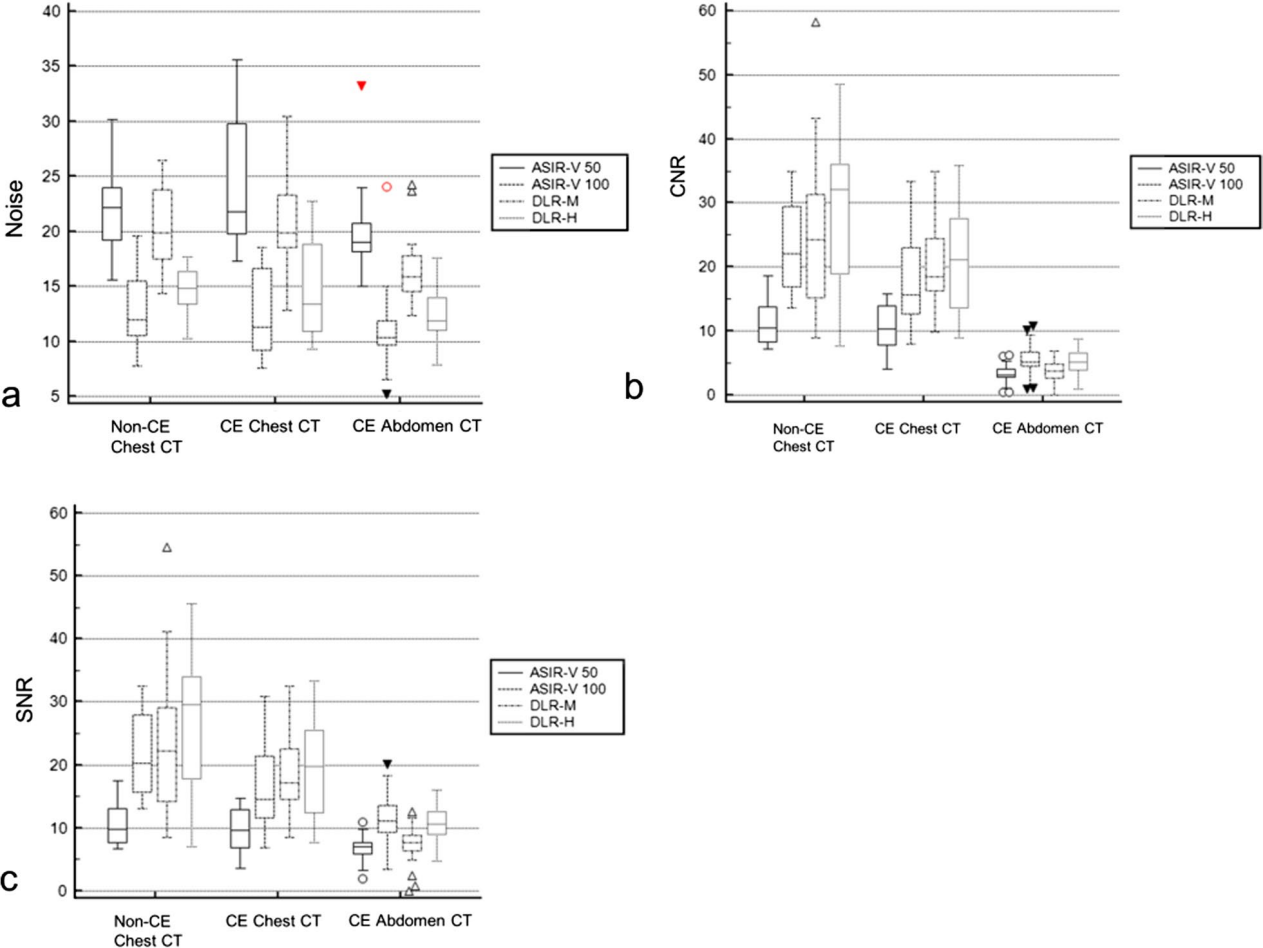
Box-and-whisker plots of quantitative pediatric CT image analyses with different reconstruction techniques. When compared with 50% adaptive statistical iterative reconstruction-V (ASIR-V), high strength deep learning reconstruction (DLR-H) was associated with **a** noise reduction, **b** better contrast to noise ratio (CNR), and **c** better signal to noise ratio (SNR). *ASIR-V 50* 50% adaptive statistical iterative reconstruction-V, *ASIR-V 100* 100% ASIR-V, *DLR-M* medium strength deep learning reconstruction, *DLR-H* high strength DLR, *CE* contrast-enhanced

The results of the quantitative image assessment are summarized in Table 1 and Fig. 1. The mean attenuation values between reconstructions were equivalent.

**Table 1:** Quantitative image analysis of pediatric CT with different reconstruction techniques in comparison with 50% ASIR-V

Parameters		ASIR-V 50	ASIR-V 100		DLR-M		DLR-H	
*Chest CT without contrast enhancement (n = 16)*								
Attenuation (HU)	lung	− 795.9 ± 91.1	− 796.2 ± 94.2	0.872	− 797.6 ± 94.3	0.872	− 796.7 ± 93.9	0.872
	paraspinal muscle	56.1 ± 11.1	54.6 ± 12.1	0.552	56.3 ± 10.7	0.552	54.9 ± 9.8	0.552
Noise		21.8 ± 3.7	15.6 ± 10.9	0.159	20.2 ± 3.8	0.455	14.6 ± 2.5	** < 0.001**
CNR	Lung	11.4 ± 3.7	22.9 ± 6.9	** < 0.001**	25.8 ± 13.3	** < 0.001**	28.4 ± 11.4	** < 0.001**
SNR	Lung	10.7 ± 3.5	21.4 ± 6.4	** < 0.001**	24.1 ± 12.5	** < 0.001**	26.6 ± 10.8	** < 0.001**
*Chest CT with contrast enhancement (n* = *12)*								
Attenuation (HU)	Lung	− 718.9 ± 139.4	− 718.7 ± 144.0	0.989	− 720.5 ± 145.4	0.989	− 719.1 ± 142.8	0.989
	Paraspinal muscle	65.0 ± 8.5	64.6 ± 5.8	0.425	64.3 ± 5.2	0.425	62.6 ± 6.8	0.425
Noise		24.5 ± 6.1	12.6 ± 3.8	** < 0.001**	21.3 ± 4.9	0.172	14.8 ± 4.7	** < 0.001**
CNR	Lung	10.4 ± 4.0	17.8 ± 8.1	**0.010**	20.0 ± 6.9	**0.001**	21.4 ± 8.6	** < 0.001**
SNR	Lung	9.6 ± 3.7	16.4 ± 7.6	**0.011**	18.4 ± 6.6	**0.001**	19.8 ± 8.1	** < 0.001**
*Abdomen CT with contrast enhancement (n* = *23)*								
Attenuation (HU)	Liver	131.4 ± 28.4	131.7 ± 28.7	0.369	125.9 ± 39.1	0.369	132.6 ± 28.6	0.369
	Aorta	185.7 ± 45.2	184.9 ± 45.9	1.000	185.3 ± 45.1	0.227	187.6 ± 45.4	0.085
	Paraspinal muscle	71.2 ± 8.4	71.3 ± 6.6	1.000	72.7 ± 6.0	0.535	71.1 ± 6.2	1.000
Noise		19.9 ± 3.7	11.1 ± 3.6	** < 0.001**	16.3 ± 3.1	**0.002**	12.2 ± 2.4	** < 0.001**
CNR	Liver	3.2 ± 1.7	5.3 ± 3.0	** < 0.001**	3.2 ± 2.4	1.000	4.9 ± 2.5	** < 0.001**
	Aorta	5.3 ± 2.2	9.5 ± 4.3	** < 0.001**	5.8 ± 1.9	**0.012**	8.0 ± 2.9	** < 0.001**
SNR	Liver	6.8 ± 2.1	11.5 ± 3.9	** < 0.001**	7.6 ± 2.7	0.583	10.7 ± 2.9	** < 0.001**
	Aorta	8.5 ± 2.4	15.6 ± 5.1	** < 0.001**	9.7 ± 2.0	**0.002**	13.0 ± 3.2	** < 0.001**

The **p**-values < 0.05 were marked as bold

Values are presented as the mean ± standard deviation

ASIR-V 50, 50% adaptive statistical iterative reconstruction-V

*ASIR-V 100* 100% ASIR-V, *DLR-M* medium strength deep learning reconstruction, *DLR-H*, high strength DLR, *CNR* contrast to noise ratio, *SNR* signal to noise ratio. Image noise is based on standard deviation of paraspinal muscle attenuation

**Fig. 2:**
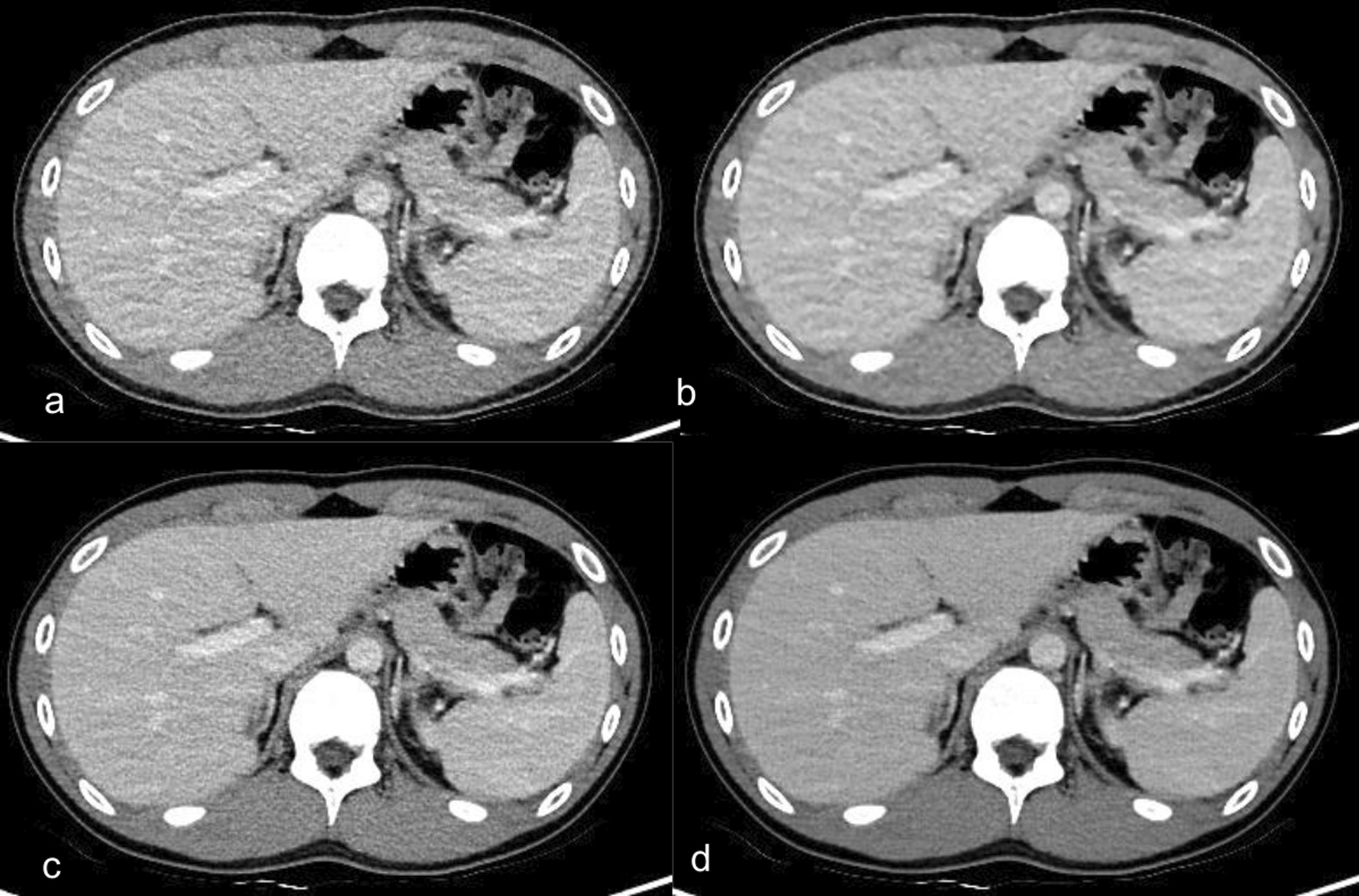
Abdomen CT images with contrast enhancement in a 15-year-old boy who had abdominal pain with a BMI of 19.9 kg/m^2^. **a**–**d** Axial contrast-enhanced CT images of the same anatomical location show image quality comparison between **a** standard 50% adaptive statistical iterative reconstruction-V (50% ASIR-V), **b** 100% ASIR-V, **c** medium-strength deep learning image reconstruction (DLR-M), and **d** high-strength deep learning image reconstruction (DLR-H). Contrast to noise ratio (CNR) in the liver was 2.18 in 50% ASIR-V, 2.84 in 100% ASIR-V, 3.03 in DLR-M, 3.88 in DLR-H

When compared with 50% ASIR-V, high strength DLR was associated with noise reduction in non-contrast chest CT (33.0%), contrast-enhanced chest CT (39.6%), and contrast-enhanced abdomen CT (38.7%) with increases in CNR at 149.1%, 105.8%, and 53.1%, respectively, and increases in SNR at 148.6%, 106.3%, and 57.4%, respectively (Fig. 2, Additional file 1: Fig. S1–S4).

Medium strength DLR also showed decreased noise in abdomen CT, but no significant difference was found in noise in chest CT when compared with 50% ASIR-V. Medium strength DLR showed better CNR and SNR in both non-contrast and contrast-enhanced chest CT; however, there was no significant difference in CNR and SNR in abdomen CT.

When compared with 100% ASIR-V, high strength DLR showed improved CNR in chest CT images without contrast enhancement by 24%. However, there was no significant improvement in CNR in both chest CT and abdomen CT images with contrast enhancement (Additional file 2: Table S1).

**Fig. 3:**
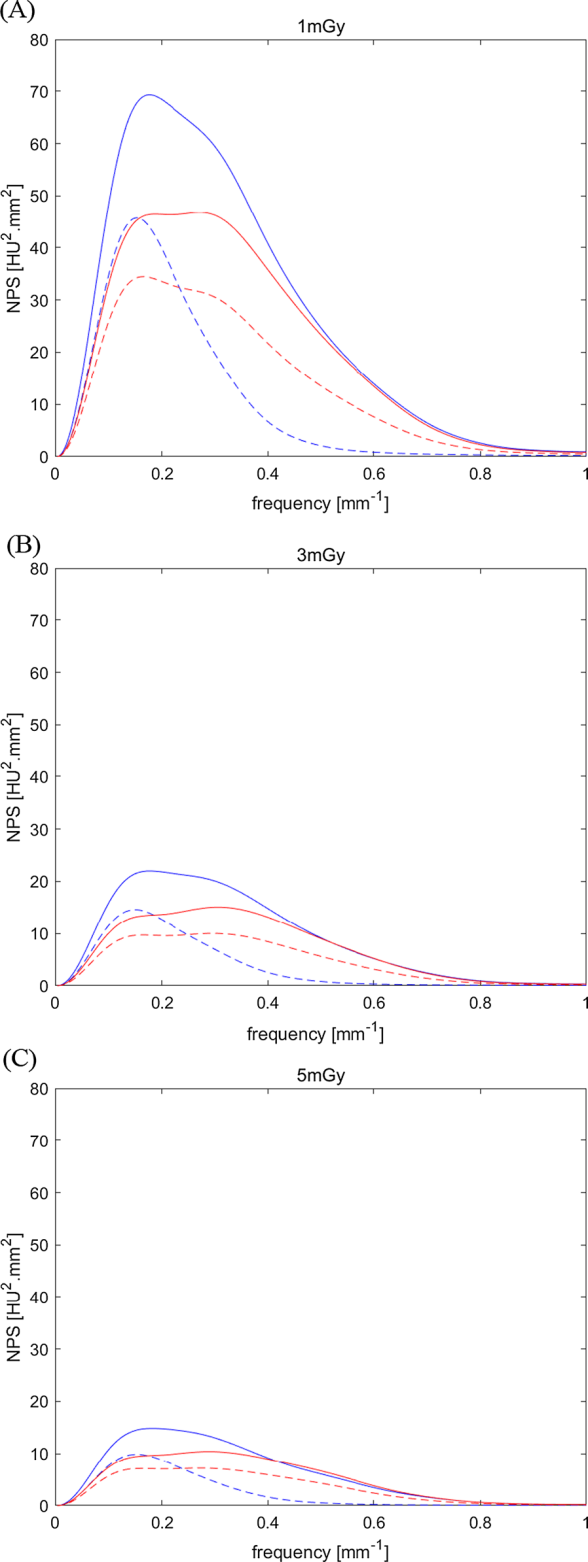
Noise power spectrum (NPS) results measured by a uniform phantom. **a**–**c** Each line represents the standard 50% adaptive statistical iterative reconstruction-V (50% ASIR-V, blue line), 100% ASIR-V (blue dotted line), medium-strength deep learning image reconstruction (DLR-M, red line), and high-strength deep learning image reconstruction (DLR-H, red dotted lines) at the dose level of **a** 1 mGy, **b** 3 mGy, and **c** 5 mGy. The NPS peaks of all reconstitution methods decreased in the order of 50% ASIR-V, DLR-M, 100% ASIR-V, and DLR-H

Figure 3 shows the NPS curves according to the clinical dose levels and image reconstruction methods, and the NPS peak and average spatial frequency for each NPS curve are summarized in Table 2. In all image reconstruction methods, as the dose increased (1 to 5 mGy), the NPS peak decreased, and the decrease rate was similar to about 21%. At the same dose level, the NPS peaks of all reconstitution methods decreased in the order of 50% ASIR-V, DLR-M, 100% ASIR-V, and DLR-H. However, the peaks of 100% ASIR-V and DLR-M were almost similar. In all image reconstruction methods, the NPS average spatial frequency showed no significant difference according to the change in dose. However, DLR methods overall showed higher average spatial frequency values than ASIR-V, and in particular, the average spatial frequency of 100% ASIR-V showed the lowest average. Overall, the DLR methods showed a pattern of remarkably reducing the magnitude of noise while maintaining the texture.

**Table 2:** Peaks and average spatial frequency of noise power spectrum (NPS) curve

Reconstruction	Dose (mGy)	ASIR-V 50	ASIR-V 100	DLR-M	DLR-H
NPS peak (HU^2^.mm^2^)	1	69.30	45.71	46.76	34.47
	3	22.03	14.61	15.10	10.03
	5	14.86	9.86	10.40	7.20
NPS average spatial frequency (mm^−1^)	1	0.27	0.19	0.30	0.28
	3	0.29	0.19	0.32	0.31
	5	0.28	0.19	0.32	0.31

*ASIR-V 50* 50% adaptive statistical iterative reconstruction-V, *ASIR-V 100* 100% ASIR-V, *DLR-M* medium strength deep learning reconstruction, *DLR-H* high strength DLR

We also analyzed the effects of body weight on noise reduction. In DLR group, the paraspinal muscle noise reduction was better in patients over 20 kg than in patients under 20 kg in both high strength group (noise: 16.9 in < 20 kg group vs. 13.3 in 20–60 kg group [p = 0.033] and 12.4 in > 60 kg group [p = 0.015]) and medium strength group (noise: 23.2 in < 20 kg group vs. 18.2 in 20–60 kg group [p = 0.028] and 17.7 in > 60 kg group [p = 0.014]). However, the noise was not different according to the body weight group in ASIR-V images.

### Qualitative image assessment

**Fig. 4:**
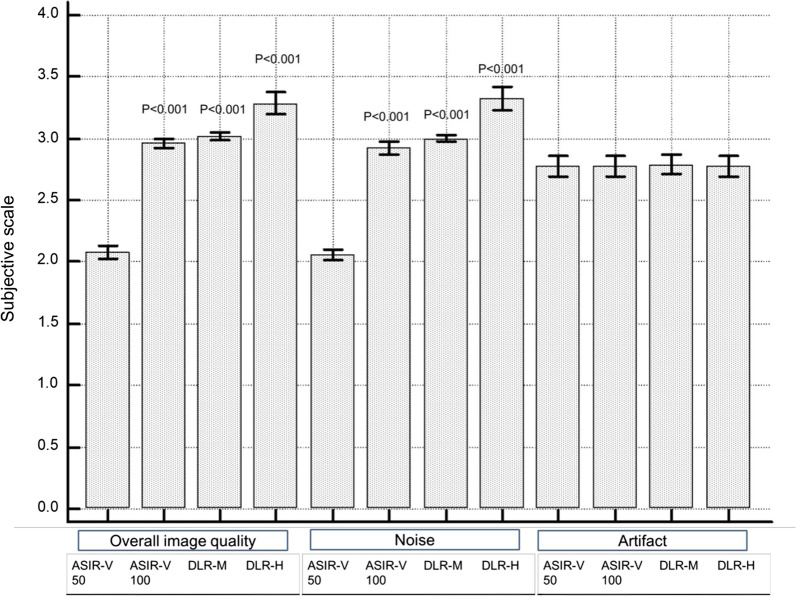
Qualitative image analysis of chest and abdomen CT from different reconstruction techniques. The four-point scale was used as follows; superior (4), average (3), suboptimal (2), unacceptable (1). Deep learning reconstruction (DLR) showed better overall image quality and noise compared with 50% adaptive statistical iterative reconstruction-V (ASIR-V); however, artifacts were not different between different reconstruction techniques. *ASIR-V 50* 50% adaptive statistical iterative reconstruction-V, *ASIR-V 100* 100% ASIR-V, *DLR-M* medium strength deep learning reconstruction, *DLR-H* high strength DLR

**Fig. 5:**
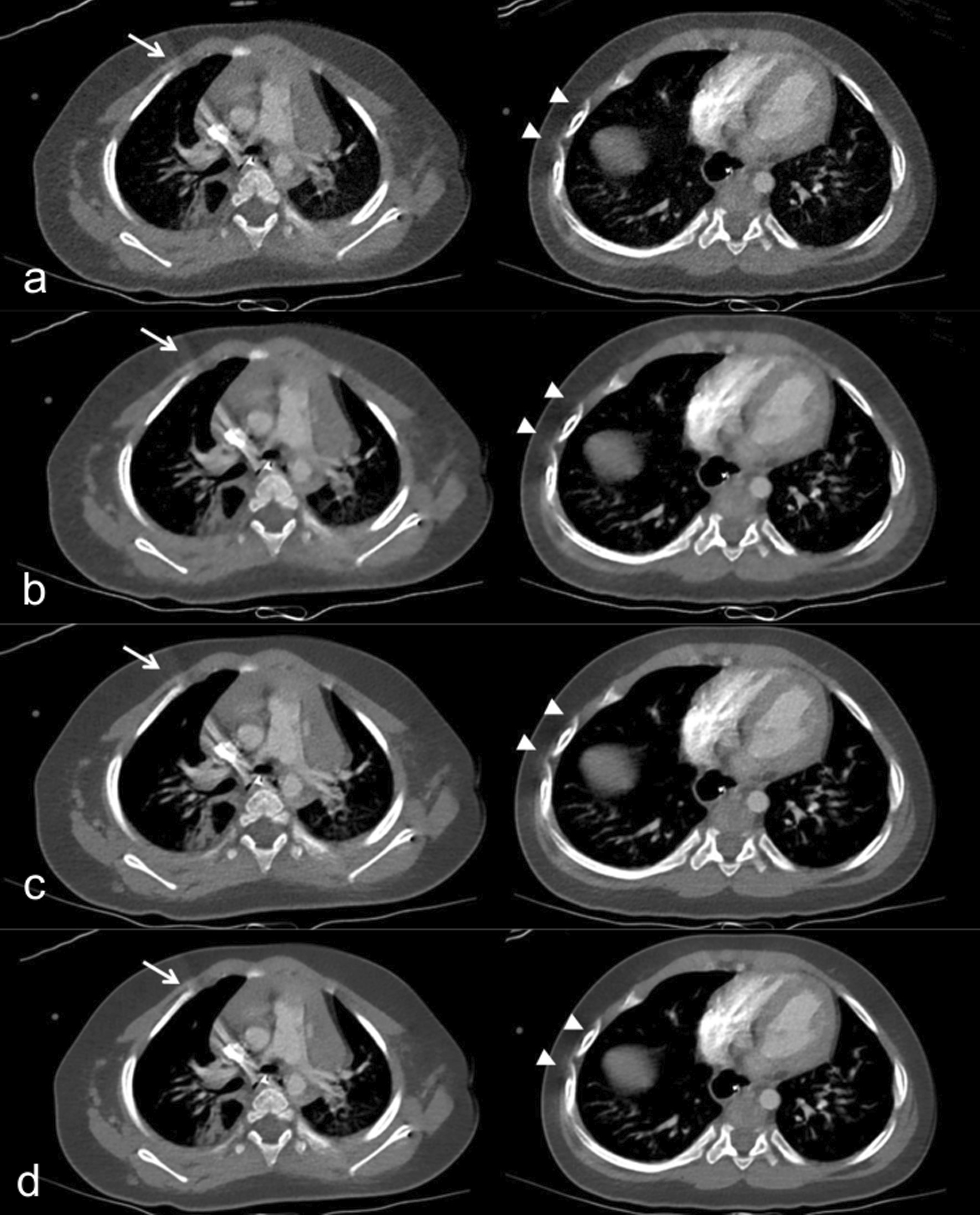
Chest CT images with mediastinal window of a 1-year-old girl who had a cough. **a**–**d** Axial contrast-enhanced CT images with **a** standard 50% adaptive statistical iterative reconstruction-V (50% ASIR-V), **b** 100% ASIR-V, **c** medium-strength deep learning image reconstruction (DLR-M), and **d** high-strength deep learning image reconstruction (DLR-H) show no difference in beam hardening artifacts due to dense contrast material in the superior vena cava (arrow) and motion artifacts in the bilateral ribs (arrow heads), resulting in lower reader scores for artifacts. Both readers thought the image was suboptimal. Contrast to noise ratio (CNR) of the lung was 4.8 in 50% ASIR-V, 12.0 in 100% ASIR-V, 13.0 in DLR-M, and 15.0 in DLR-H

The results of the subjective image quality analyses are summarized in Table 3 and Fig. 4. The subjective assessment of overall image quality and noise were also better on DLR images both on medium and high strength compared to 50% ASIR-V (*p* < 0.001). The agreement was moderate for overall image quality and good for noise in high strength DLR (*p* < 0.001). However, there was poor agreement in both image quality and noise in medium strength DLR (*p* < 0.001). There was no significant difference in motion or beam hardening artifacts between reconstruction methods with an excellent interobserver agreement (κ = 0.944, *p* < 0.001) (Fig. 5).

**Table 3:** Distribution of subjective image scoring for different reconstruction techniques by two pediatric radiologists

Parameter	Reviewer 1	Reviewer 2	*Agreement (κ)
*Overall image quality*			
(1/2/3/4)			
ASIR-V 50	0/47/4/0	0/47/4/0	0.728
ASIR-V 100	0/4/47/0	0/0/51/0	0
DLR-M	0/0/49/1	0/0/49/1	-0.02
DLR-H	0/0/38/13	0/0/35/16	0.568
*Noise*			
(1/2/3/4)			
ASIR-V 50	0/47/4/0	0/49/2/0	0.297
ASIR-V 100	0/8/43/0	0/0/51/0	0
DLR-M	0/1/48/1	0/0/51/0	0
DLR-H	0/1/34/16	0/0/33/18	0.62
*Artifact*			
(1/2/3/4)			
ASIR-V 50	0/11/40/0	0/12/39/0	0.944
ASIR-V 100	0/11/40/0	0/12/39/0	0.944
DLR-M	0/11/40/0	0/12/39/0	0.94
DLR-H	0/11/40/0	0/12/39/0	0.944

Four-point scale: 4 superior, 3 average, 2 suboptimal, and 1 unacceptable

*ASIR-V 50* 50% adaptive statistical iterative reconstruction-V, *ASIR-V 100* 100% ASIR-V, *DLR-M* medium strength deep learning reconstruction, *DLR-H* high strength DLR

## Discussion

Our study found that DLR can improve the quantitative and qualitative image quality in pediatric chest and abdomen CT relative to advanced IR technique, our standard 50% ASIR-V. High-strength DLR showed significant noise reduction with increased CNR and SNR. DLR also scored significantly better for image quality and noise subjectively. However, motion or beam hardening artifacts were not decreased with deep learning method, regardless of strength.

There have been efforts to improve image quality of low dose CT imaging by decreasing noise and artifacts with various reconstruction methods [25–27]. Recently, the DLR algorithm has been developed for CT to remove image noise. The effect of DLR on image quality and its potential to lower patient radiation dose is being investigated. A phantom study demonstrated that DLR had superior noise, magnitude, noise texture, and spatial resolution [11]. Another study also showed that DLR improves the image quality through noise reduction and increased CNR without altering the image texture on abdomen CT [12]. They demonstrated that subjective diagnostic confidence was increased in all DLR images when compared with ASIR-V with a 30% blending factor, and the higher strength in DLR lowers the noise with increased sharpness [13]. The SNR and CNR values of high-strength DLR images were higher than those of ASIR-V with 80 or 100% blending factor. Similar results were also reported in studies with different vendor systems and algorithms [10, 14, 15].

DLR has been introduced to pediatric patients in a few studies of abdomen CT [16–18]. Lim et al. [16] studied a 5-year-old patient’s phantom and pediatric abdomen CT exams using a vendor-neutral DLR technique and demonstrated similar image quality with a hybrid IR technique. Brady et al. [17] used contrast-enhanced abdomen CT with DLR algorithm showing improved object detectability, reduced image noise, and high radiologist preference when compared to conventional IR images. About a 51% dose reduction using DLR was hypothesized based on mathematical extrapolation from this retrospective study. Lee et al. [18] used DLR with low iodine concentration abdominal dual-energy CT and showed decreased noise in DLR images without difference in CNR, overall image quality, and diagnostic quality of lesions. The CTDI_vol_ and total iodine administration were lower in dual energy CT with DLR. Both studies suggested that DLR has the potential to improve image quality and potentially reduce patient radiation dose. However, no study has evaluated the role of DLR in pediatric chest CT and the effect of DLR on image artifacts.

Our study shows similar results in noise reduction and quality improvement. High strength DLR was associated with noise reduction in non-contrast chest CT, contrast-enhanced chest CT, and contrast-enhanced abdomen CT with an increase in both CNR and SNR. The subjective assessment of overall image quality and noise were also better on DLR images both on medium and high strength DLR compared to 50% ASIR-V. Our study showed no significant difference in attenuation values of the organs in pediatric chest and abdomen. This result is comparable with a previous report with an adult population [12]. Therefore, we can use CT images with DLR for attenuation analyses such as emphysema index measurements.

Previous studies have focused on noise reduction and image quality improvement of DLR with little focus on artifacts. DLR scored better on artifacts than 30% ASIR-V images in a previous study [12]. Another study reported no DLR related image artifacts [14]. A prior study has reported more frequent distortion artifacts with DLR [28]. In our study, there was no significant difference in artifacts between reconstruction methods with excellent inter-observer agreement on artifacts. Mainly these artifacts were beam hardening artifacts from metal or dense contrast media in vessels. The motion and beam hardening artifact reduction were not significant by TrueFidelity in our study. This may be due to a lack of learning about these artifacts and may suggest that TrueFidelity is weak in this perspective. Future learning about these artifacts may be required for better image reconstruction. However, unlike previous study, there was no significant distortion artifacts in our study. Depending on the purpose and input data of the DLR technology, the role of DLR may vary. It would be better if DLR algorithm is developed as an open source so that it can be used in various equipment and undergo further development by other researchers.

Our study has limitations. First, the sample size of our retrospective study was small, and we could not evaluate lesion detectability or diagnostic accuracy. Second, the data is from a designated vendor’s DLR algorithm. Since it was hard to get the projection data from the vendors directly, we could not compare other DLR, such as the image-domain-based method. Third, the number of patients with artifacts was not the majority of the patient population. Fourth, from the retrospective nature of our study, we could not compare images between FBP and DLR. Fifth, our study cannot suggest an estimated radiation dose reduction using DLR. Additional prospective studies with more patients are needed.

## Conclusions

Compared with 50% ASIR-V, DLR improved the CT evaluation of pediatric chest and abdomen images with significant noise reduction. However, motion or beam hardening artifacts were not decreased by DLR, regardless of strength.

## Supplementary Information

**Additional file 1.** Fig. S1–S4: Abdomen and chest CT images with 50% ASIR-V, 100% ASIR-V, DLR-M and DLR-H for image comparison.

**Additional file 2. Table S1**: Quantitative image analysis of pediatric CT with different reconstruction techniques in comparison with 100% ASIR-V.

## Data Availability

All data generated or analyzed during this study are included in this published article and its supplementary figures and table. The datasets used and analyzed during the current study are available from the corresponding author on reasonable request.

## Abbreviations

CT: Computed tomography
DLR: Deep learning reconstruction
IR: Iterative reconstruction
ASIR-V: Adaptive statistical iterative reconstruction V
CNR: Contrast to noise ratio
SNR: Signal to noise
SD: Standard deviation
NPS: Noise power spectrum
ANOVA: Analysis of variance
FBP: Filtered back projection
CTDI vol: CT dose index volume
SSDE: Size-specific dose estimates
DLP: Dose-length product
ED: Effective dose
