# Supplementary material for: Image quality assessment of pediatric chest and abdomen CT by deep learning reconstruction
Source: BMC Med Imaging. 2021 Oct 10;21:146. doi: 10.1186/s12880-021-00677-2 (PMC8503996; doi:10.1186/s12880-021-00677-2)

Supplementary figure 1.

Abdomen CT images with contrast enhancement in a 16-year-old girl who had abdominal pain with a BMI of 15.2 kg/m^2^.

(A-D) Axial contrast-enhanced CT images of the same anatomical location show image quality comparison between (A) standard 50% adaptive statistical iterative reconstruction-V (50% ASIR-V), (B) 100% ASIR-V, (C) medium-strength deep learning image reconstruction (DLR-M), and (D) high-strength deep learning image reconstruction (DLR-H). Contrast to noise ratio (CNR) in the liver was 6.04 in 50% ASIR-V, 10.19 in 100% ASIR-V, 6.97 in DLR-M, 8.83 in DLR-H. While some DLR-H had lower CNR than 100% ASIR-V in abdomen, there was less blurring and more standard dose-like margin in the DLR-H images.


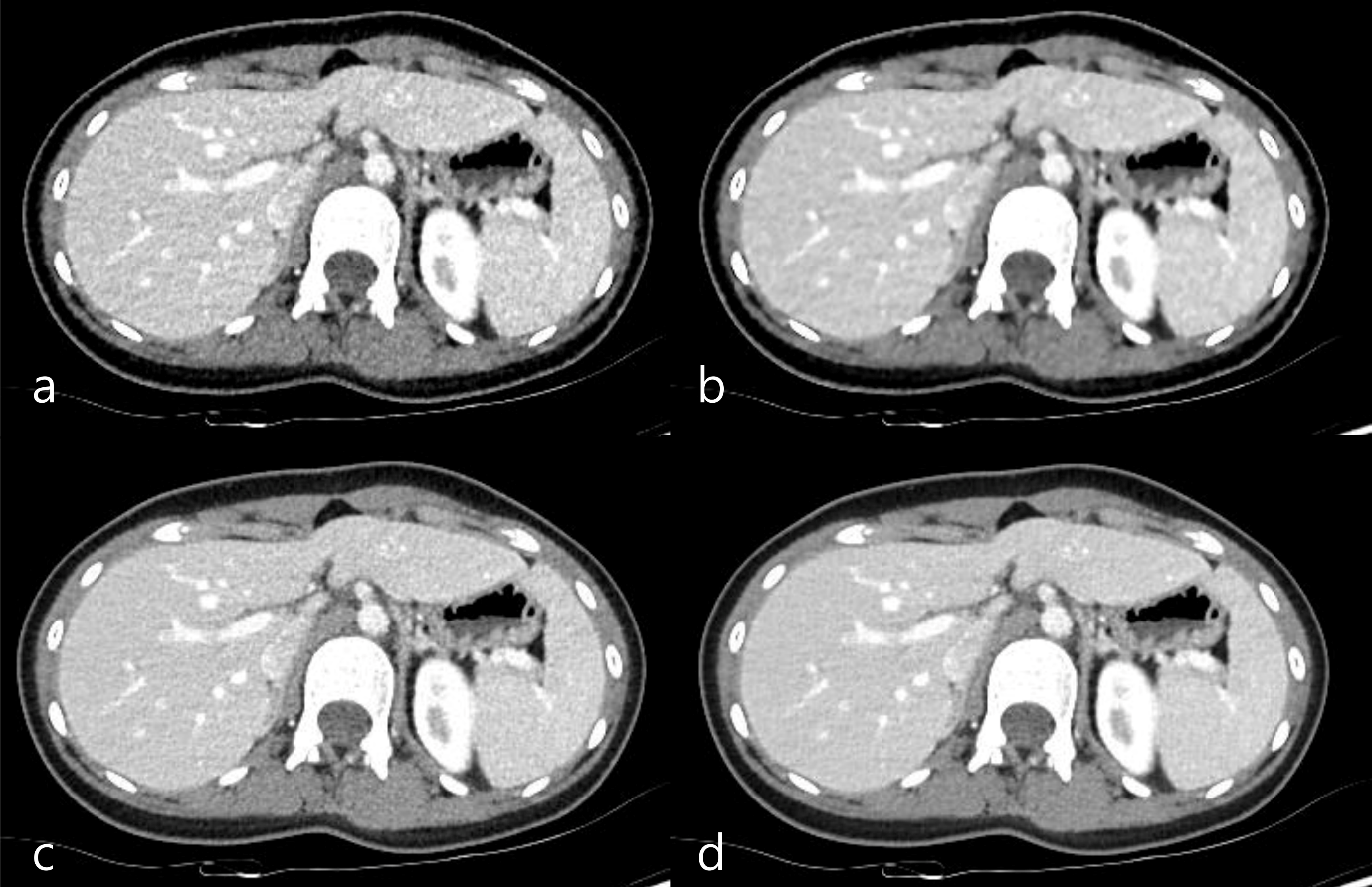


Supplementary figure 2.

Abdomen CT images with contrast enhancement in a 15-year-old boy who had malignant melanoma with multiple liver metastasis and BMI of 14.5 kg/m^2^.

(A-D) Axial contrast-enhanced CT images of the same anatomical location show image quality comparison between (A) 50% ASIR-V, (B) 100% ASIR-V, (C) DLR-M, and (D) DLR-H. Contrast to noise ratio (CNR) in the liver was 2.77 in 50% ASIR-V, 4.72 in 100% ASIR-V, 3.73 in DLR-M, 5.29 in DLR-H. The multiple liver metastases are shown more naturally with clear margin in the DLR-H image.


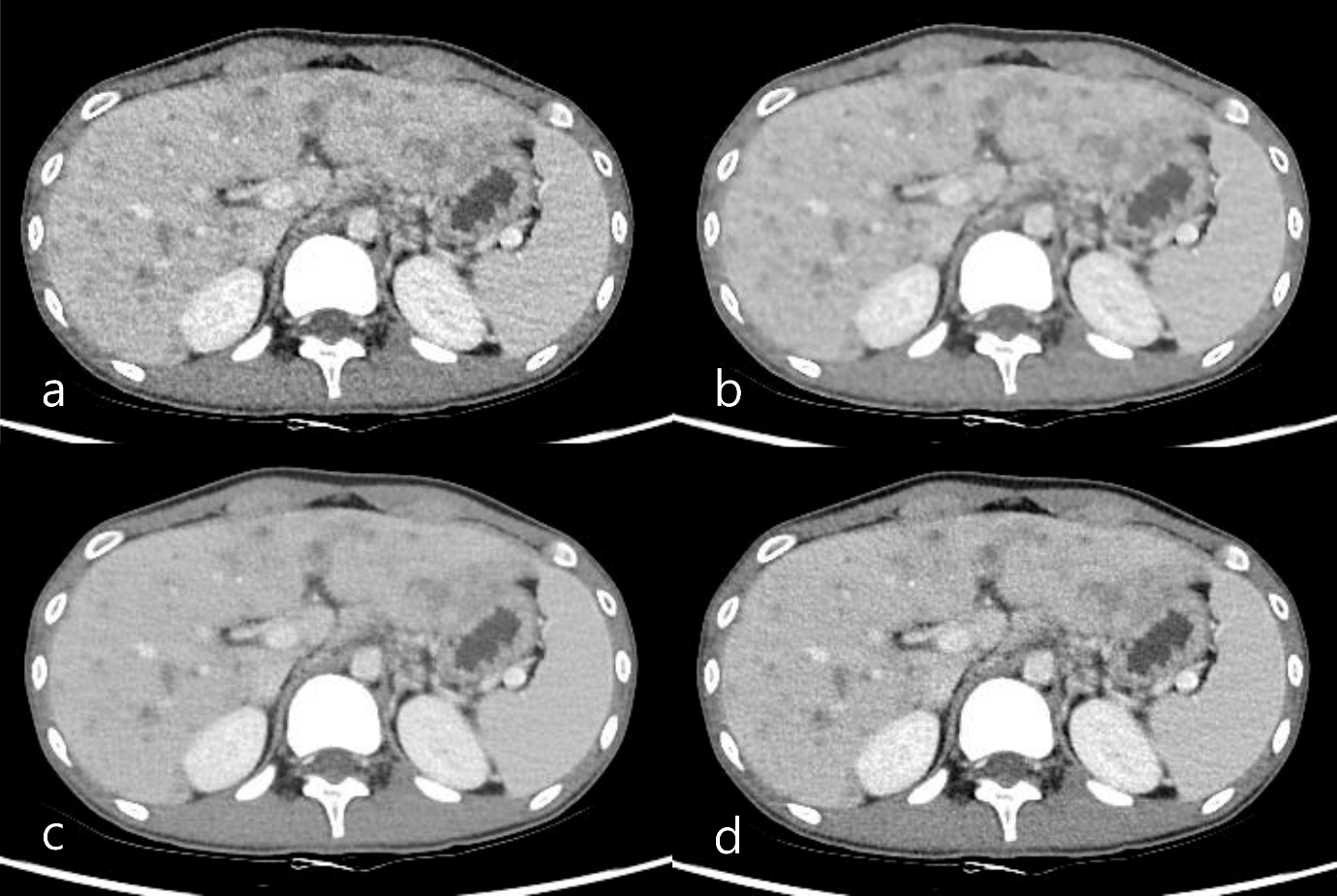


Supplementary figure 3

Chest CT images with contrast enhancement in a 16-year-old boy who had metastatic undifferentiated carcinoma at cervical spine with suspicious lung metastasis and BMI of 22.7 kg/m^2^.

(A-D) Axial contrast-enhanced CT images of the same anatomical location show image quality comparison between (A) 50% ASIR-V, (B) 100% ASIR-V, (C) DLR-M, and (D) DLR-H. Contrast to noise ratio (CNR) in the lung was 15.59 in 50% ASIR-V, 27.24 in 100% ASIR-V, 22.06 in DLR-M, 30.65 in DLR-H. The tiny lung metastasis at left upper lobe is shown with clear margin in the DLR-H image (arrow).


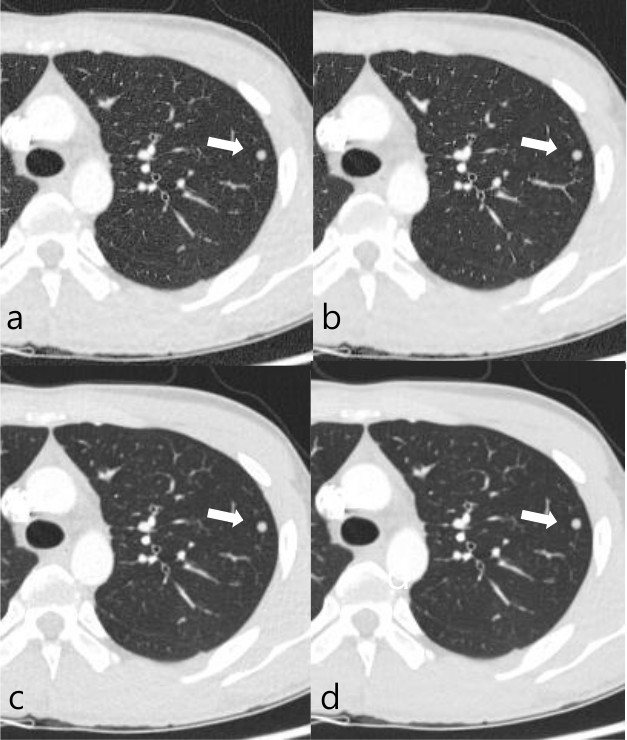


Supplementary figure 4

Chest CT images with contrast enhancement in a 15-year-old boy with BMI of 24.2 kg/m^2^ who had palpable lesion in left chest wall which was confirmed as nodular fasciitis.

(A-D) Axial contrast-enhanced CT images of the same anatomical location show image quality comparison between (A) 50% ASIR-V, (B) 100% ASIR-V, (C) DLR-M, and (D) DLR-H. Contrast to noise ratio (CNR) in the lung was 13.47 in 50% ASIR-V, 16.90 in 100% ASIR-V, 19.86 in DLR-M, 20.21 in DLR-H. The nodular enhancing lesion at left lateral chest wall are shown with clear margin in the DLR-H image (arrow).


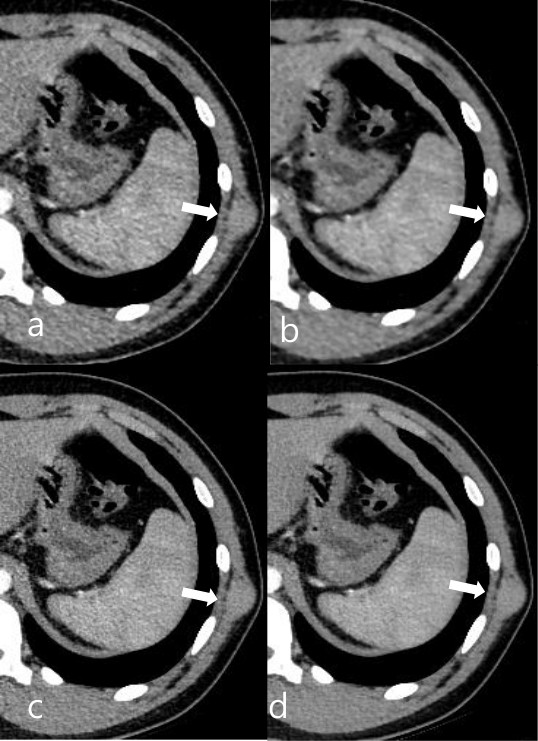

Supplement: Supplementary file 1 — Additional file 1. Fig. S1–S4: Abdomen and chest CT images with 50% ASIR-V, 100% ASIR-V, DLR-M and DLR-H for image comparison. [file 12880_2021_677_MOESM1_ESM.docx]
