# Supplementary material for: Image quality assessment of pediatric chest and abdomen CT by deep learning reconstruction
Source: BMC Med Imaging. 2021 Oct 10;21:146. doi: 10.1186/s12880-021-00677-2 (PMC8503996; doi:10.1186/s12880-021-00677-2)
Supplement: Supplementary file 2 — Additional file 2. Table S1: Quantitative image analysis of pediatric CT with different reconstruction techniques in comparison with 100% ASIR-V. [file 12880_2021_677_MOESM2_ESM.docx]

**Supplementary material 1.**

Quantitative image analysis of pediatric CT with different reconstruction techniques in comparison with 100% ASIR-V

| Parameters | | ASIR-V 50 | | ASIR-V 100 | DLR-M | | DLR-H | |
| --- | --- | --- | --- | --- | --- | --- | --- | --- |
| Chest CT without contrast enhancement (n=16) | | | | | | | | |
| Attenuation (HU) | lung | -795.9 ± 91.1 | 0.872 | -796.2 ± 94.2 | -797.6 ± 94.3 | 0.872 | -796.7 ± 93.9 | 0.872 |
|  | paraspinal muscle | 56.1 ± 11.1 | 0.552 | 54.6 ± 12.1 | 56.3 ± 10.7 | 0.552 | 54.9 ± 9.8 | 0.552 |
| Noise | | 21.8 ± 3.7 | 0.160 | 15.6 ± 10.9 | 20.2 ± 3.8 | 0.759 | 14.6 ± 2.5 | 1.000 |
| CNR | lung | 11.4 ± 3.7 | **<0.001** | 22.9 ± 6.9 | 25.8 ± 13.3 | **<0.001** | 28.4 ± 11.4 | **<0.001** |
| SNR | lung | 10.7 ± 3.5 | **<0.001** | 21.4 ± 6.4 | 24.1 ± 12.5 | 1.000 | 26.6 ± 10.8 | 0.118 |
| Chest CT with contrast enhancement (n=12) | | | | | | | | |
| Attenuation (HU) | lung | -718.9 ± 139.4 | 0.989 | -718.7 ± 144.0 | -720.5 ± 145.4 | 0.989 | -719.1 ± 142.8 | 0.989 |
|  | paraspinal muscle | 65.0 ± 8.5 | 0.425 | 64.6 ± 5.8 | 64.3 ± 5.2 | 0.425 | 62.6 ± 6.8 | 0.425 |
| Noise | | 24.5 ± 6.1 | **<0.001** | 12.6 ± 3.8 | 21.3 ± 4.9 | **<0.001** | 14.8 ± 4.7 | 0.319 |
| CNR | lung | 10.4 ± 4.0 | **0.010** | 17.8 ± 8.1 | 20.0 ± 6.9 | 1.000 | 21.4±8.6 | 0.847 |
| SNR | lung | 9.6 ± 3.7 | **0.010** | 16.4 ± 7.6 | 18.4 ± 6.6 | 1.000 | 19.8±8.1 | 0.850 |
| Abdomen CT with contrast enhancement (n=23) | | | | | | | | |
| Attenuation (HU) | liver | 131.4 ± 28.4 | 0.369 | 131.7 ± 28.7 | 125.9 ± 39.1 | 0.369 | 132.6 ± 28.6 | 0.369 |
|  | aorta | 185.7 ± 45.2 | 1.000 | 184.9 ± 45.9 | 185.3 ± 45.1 | 0.131 | 187.6 ± 45.4 | **0.017** |
|  | paraspinal muscle | 71.2 ± 8.4 | 1.000 | 71.3 ± 6.6 | 72.7 ± 6.0 | 0.638 | 71.1 ± 6.2 | 1.000 |
| Noise | | 19.9 ± 3.7 | **<0.001** | 11.1 ± 3.6 | 16.3 ± 3.1 | **<0.001** | 12.2 ± 2.4 | **0.038** |
| CNR | liver | 3.2 ± 1.7 | **<0.001** | 5.3 ± 3.0 | 3.2 ± 2.4 | **<0.001** | 4.9 ± 2.5 | 0.687 |
|  | aorta | 5.3 ± 2.2 | **<0.001** | 9.5 ± 4.3 | 5.8 ± 1.9 | **<0.001** | 8.0 ± 2.9 | 0.633 |
| SNR | liver | 6.8 ± 2.1 | **<0.001** | 11.5 ± 3.9 | 7.6 ± 2.7 | **<0.001** | 10.7 ± 2.9 | **0.004** |
|  | aorta | 8.5 ± 2.4 | **<0.001** | 15.6 ± 5.1 | 9.7 ± 2.0 | **0.002** | 13.0 ± 3.2 | **<0.001** |

Note: Values are mean ±SD. CNR= contrast to noise ratio. SNR= signal to noise ratio, ASIR-V 50 = 50% adaptive statistical iterative reconstruction V, DLR = deep learning image reconstruction at medium, and high strengths. Mean image noise is based on paraspinal muscle and is calculated as the mean ± SD of attenuation in Hounsfield units.
